# High Abundance and Genetic Variability of Atypical Porcine Pestivirus in Pigs from Europe and Asia

**DOI:** 10.3201/eid2312.170951

**Published:** 2017-12

**Authors:** Alexander Postel, Denise Meyer, Gökce Nur Cagatay, Francesco Feliziani, Gian Mario De Mia, Nicole Fischer, Adam Grundhoff, Vesna Milićević, Ming-Chung Deng, Chia-Yi Chang, Hua-Ji Qiu, Yuan Sun, Michael Wendt, Paul Becher

**Affiliations:** University of Veterinary Medicine, Hannover, Germany (A. Postel, D. Meyer, G.N. Cagatay, M. Wendt, P. Becher);; Istituto Zooprofilattico Sperimentale dell’Umbria e delle Marche, Perugia, Italy (F. Feliziani, G.M. De Mia);; University Medical Center Hamburg-Eppendorf, Hamburg, Germany (N. Fischer);; Heinrich Pette Institute, Hamburg (A. Grundhoff);; Institute of Veterinary Medicine of Serbia, Belgrade, Republic of Serbia (V. Milićević);; Animal Health Research Institute, New Taipei City, Taiwan (M.-C. Deng, C-Y. Chang);; Harbin Veteri-nary Research Institute, Harbin, China (H.-J. Qiu, Y. Sun)

**Keywords:** atypical porcine pestivirus, Europe, Asia, genome detection, serology, genetic variability, viruses, zoonoses, geographic distribution

## Abstract

Atypical porcine pestivirus (APPV) was recently reported to be associated with neurologic disorders in newborn piglets. Investigations of 1,460 serum samples of apparently healthy pigs from different parts of Europe and Asia demonstrate a geographically wide distribution of genetically highly variable APPV and high APPV genome and antibody detection rates.

Pestiviruses are highly variable RNA viruses within the family *Flaviviridae*. The recently discovered atypical porcine pestivirus (APPV) is capable of inducing neurologic disorder in its host, like other pathogens of this family (e.g., tick-borne encephalitis virus, Zika virus). Several recently published reports demonstrate that APPV is a prominent cause of virus-induced congenital tremor in pigs (*1**–**4*). Serum samples from healthy but viremic animals can induce birth of clinically affected offspring when experimentally transferred to sows during gestation (*1**,**2*). So far, APPV presence has been reported from the United States, some countries within Europe, and China (*2**,**4**–**7*). The economic relevance of APPV-related losses in pig production remains to be determined, but estimation revealed a drop in reproductive performance of 10% in an affected farm (*4*). Early data from the United States and Germany suggested a relatively high abundance (2.4%–22%) of APPV genomes in apparently healthy pigs (*3**,**6**,**8*) that likely play an important epidemiologic role as virus carriers. We investigated APPV genome and antibody abundance in healthy pigs from different parts of Europe and Asia. To provide insight into genetic diversity of this novel pathogen,

We tested 1,460 serum samples from Germany, Great Britain, Italy, Serbia, Switzerland, mainland China, and Taiwan by using an APPV-specific PCR and an indirect APPV ELISA, as previously described (*3**,**9*). The sample set comprised 369 serum samples from Germany that were previously screened for the presence of APPV genomes (*3*). For our study, serum samples were taken from apparently healthy pigs within the framework of national veterinary health management in concordance with national legal and ethical regulations.

For APPV genome detection, we conducted a PCR targeting the nonstructural protein (NS) 3 encoding region and confirmed specificity of amplification by gel electrophoresis (*3*). We detected APPV genomes in domestic pigs from all investigated regions. In total, 130 (8.9%) of the 1,460 tested samples were APPV genome positive (Figure). Genome detection rates ranged from 2.3% (2/86 samples from Great Britain) to 17.5% (35/200 samples from Italy). Moreover, we demonstrated that APPV was abundant in Asia; we detected the APPV genome in 11/219 samples (5%) from mainland China and 22/200 samples (11%) from Taiwan.

**Figure:**
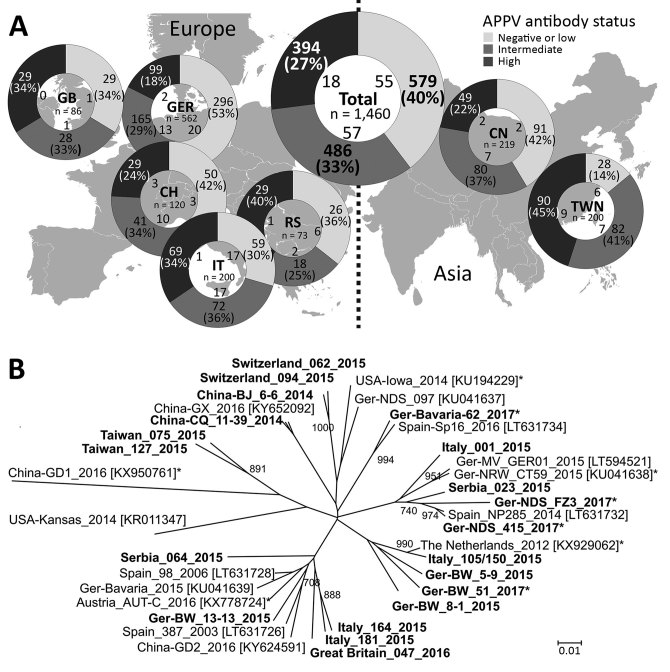
Detection rates of APPV genome and antibodies and genetic variability in Europe and Asia. A) APPV antibody status in pigs from parts of Europe and Asia. The region of origin, the number of investigated samples, and the absolute numbers of APPV genome–positive samples in dependence on the serologic category (low, intermediate, or high APPV antibody status) are shown in the central circle. B) Phylogenetic tree based on a 400-nt fragment in the nonstructural protein 3 encoding region. We calculated genetic distances using the Kimura 2-parameter model. We performed phylogenetic analysis by the neighbor-joining method including 1,000 iterations for bootstrap analysis. Only bootstrap values ≥700 are indicated. Bold indicates sequences generated in this study; asterisks indicate sequences from piglets with congenital tremor. Accession numbers for reference sequences from GenBank are shown in brackets. APPV, atypical porcine pestivirus; CH, Switzerland; CN, China; GB, Great Britain; GER, Germany; IT, Italy; RS, Serbia; TWN, Taiwan. Scale bar indicate nucleotide substitutions per site.

We used individual samples with high genome loads to generate amplicons in a seminested PCR and subsequently performed Sanger sequencing (FlexiRun, LGC Genomics, Berlin, Germany). We generated 20 different APPV NS3 sequences from apparently healthy pigs of all countries (sequences deposited into GenBank under accession nos. MF279213–32). Genetic differences reflect geographic origin to a low degree (Figure); genetic variability even within a country is remarkably high (e.g., Germany and Italy). Genetic analyses including sequence data obtained from samples of diseased piglets revealed no correlation of pathogenicity with certain genetic variants (Figure).

In addition to the NS3 fragments, we determined APPV complete coding sequences (CDS) from 1 sample from a healthy pig from China (deposited into GenBank under accession no. MF167292) and 2 samples (accession nos. MF167290 and MF167291) obtained from pigs during outbreaks of congenital tremors in Germany (*3**,**9*). We performed next-generation sequencing as previously described (*3*). The outbreak isolates from Germany were almost identical (0.2% genetic distance) and were similar to an isolate from northern Germany (accession no. LT594521). The APPV from China had a unique 93-nt deletion in the NS5A encoding region. A similar genome (97.9% identity) is lacking this deletion (Guangxi Province; accession no. KY652092). The biological relevance of the deletion remains elusive, but classical pestiviruses show a remarkable genetic tolerance in this genomic region (*10*). The sequence data we obtained reveal a high genetic variability (up to 21% genetic distance), which is comparable to that of classical swine fever virus (Technical Appendix).

We applied an indirect APPV E^rns^ antibody ELISA, as described (*9*), and classified the serologic status into low (S/p≤0.5), intermediate (0.5<S/p<1.0), or highly (S/p≥1.0) reactive. Due to the lack of reference material and a standard assay, we could not determine test parameters (e.g., sensitivity, specificity) at this stage. Nevertheless, the ELISA was a valuable tool for detecting seroconversion in infected pigs; >60% of the animals showed intermediate to high reactivity in the antibody ELISA (Figure), which is in line with high APPV genome detection rates. We detected similar frequencies of APPV antibody–positive samples for each region, independent of the genome detection rates (Figure). We found most of the viral genomes (≈86%) in samples with intermediate or low antibody status; few (≈14%) of the highly antibody-positive animals were viremic at the same time. This observation might indicate a degree of protection provided by the induced antibodies. Of the 40% of the pigs that were antibody negative, 10% were genome positive; possible explanations are that serum samples were taken either from acutely infected animals before induction of a detectable antibody response or from persistently infected animals lacking a specific humoral immune response due to a specifically acquired immunotolerance, a well-known consequence of intrauterine pestivirus infections.

Our findings indicate that the recently discovered APPV is abundant on several continents. APPV must be regarded as a pig pathogen of likely worldwide relevance.

Technical Appendix Additional information from genetic analysis of atypical porcine pestivirus in animals from Europe and Asia
