# Supplementary material for: High Abundance and Genetic Variability of Atypical Porcine Pestivirus in Pigs from Europe and Asia
Source: Emerg Infect Dis. 2017 Dec;23(12):2104–7. doi: 10.3201/eid2312.170951 (PMC5708225; doi:10.3201/eid2312.170951)
Supplement: Technical Appendix — Additional information from genetic analysis of atypical porcine pestivirus in animals from Europe and Asia [file 17-0951-Techapp-s1.pdf]

Article DOI: https://doi.org/10.3201/eid2312.170951

Technical Appendix

Technical Appendix Table. Genetic distances (in %) of APPV obtained from different geographic regions*

Region	USA-Kansas	China-CQ	Austria	China-GD2	Ger-Bavaria	Ger-NRW	USA-Iowa	Netherlands
China-GD1	19.2 (19.7)	19.1 (20.0)	20.2 (19.7)	21.3 (19.6)	20.8 (19.5)	20.9 (19.5)	18.8 (20.1)	18.1 (19.9)
USA-Kansas	–	13.9 (13.8)	13.2 (13.4)	14.9 (13.2)	12.6 (13.2)	12.3 (13.4)	13.5 (13.7)	14.8 (13.6)
China-CQ		-	9.5 (10.6)	9.8 (10.4)	10.4 (10.4)	9.8 (10.7)	10.1 (10.9)	8.9 (10.6)
Austria			-	6.1 (7.4)	3.6 (7.2)	8.9 (10.7)	11.0 (10.7)	8.9 (10.2)
China-GD2				-	5.5 (6.9)	10.7 (10.2)	11.7 (10.4)	9.5 (9.8)
Ger-Bavaria					-	11.3 (10.3)	11.6 (10.5)	10.1 (10.1)
Ger-NRW						-	10.1 (10.3)	9.5 (9.8)
USA-Iowa							-	8.9 (9.5)

*Genetic distances (Kimura 2-parameter substitution model) of a fragment in the NS3 encoding region were compared with distances among complete coding sequences (indicated by numbers in parentheses). Reference sequences were obtained from GenBank (KX950761, KR011347, KX778724, KY624591, KU041639, KU194229). CDS of APPV Ger-NRW_L277 (MF167291) and China-CQ (MF167292) were obtained within this study. Highly similar sequences harboring more that 95% genetic similarity were excluded from analysis. CQ, Chongqing; GD, Guangdong; Ger, Germany; NRW, North Rhine Westphalia.
